# Nutritional Intake and Chronicity Associated with the Old World Cutaneous Leishmaniasis: Role of Vitamin A

**Published:** 2020-01

**Authors:** Vahid MASHAYEKHI GOYONLO, Abdolreza NOROUZY, Mohsen NEMATI, Pouran LAYEGH, Saeed AKHLAGHI, Ahmad Reza TAHERI, Bita KIAFAR

**Affiliations:** 1 Cutaneous Leishmaniasis Research Center, Mashhad University of Medical Sciences, Mashhad, Iran; 2 Biochemistry of Nutrition Research Center, Faculty of Medicine, Mashhad University of Medical Sciences, Mashhad, Iran; 3 Deputy of Research, Faculty of Medicine, Mashhad University of Medical Sciences, Mashhad, Iran

**Keywords:** Cutaneous leishmaniasis, Nutrition, Vitamin A

## Abstract

**Background:**

Old world cutaneous leishmaniasis (CL) is known as a self-healing cutaneous parasitic infection. Host immunity has a fundamental role in the course of this infection. This study was designed to investigate the relationship between nutritional status and vitamin A intake with the clinical course of CL.

**Methods:**

Overall, 250 patients with CL attending a dermatology clinic in Imam Reza Hospital Mashhad, Iran, were enrolled from Apr 2011 to Aug 2012. For data gathering, a semi-quantitative 302-item food frequency questionnaire was utilized. They received routine treatment protocols for leishmaniasis and 1 year of follow-up

**Results:**

As for the 149 patients who completed the study, a deficiency of macro and micronutrients, particularly vitamin A, was significantly related to a chronic clinical disease course.

**Conclusion:**

Imbalanced or insufficient nutritional intake including vitamin A deficiency, may influence the clinical course of CL.

## Introduction

Cutaneous leishmaniasis (CL) is the clinical outcome of infection with the dermotropic species of the Leishmania parasite(1). The incidence rate is 1.5 million new cases per year, and as many as 90% of these cases occur in 6 developing countries, including Iran. Mashhad, a metropolis in the province of Khorasan Razavi, in northeast Iran, is an endemic focus for the *Leishmania tropica* form of Old World CL (2). These dermotropic species most commonly cause localized cutaneous lesions with a self-healing course within 6–12 months (3). Chronic CL or lupoid leishmaniasis is a non-healing and potentially scarring lesion caused by *L. tropic* that is resistant to treatment with all anti-leishmanial treatment modalities. Although there is no widely accepted definition for acute versus chronic cutaneous leishmaniasis most experts agree on a time boundary of twelve months (4,5).

Briefly, clinical expression and severity of the disease are determined by the interplay between the parasite and the host's immune system, ranging from asymptomatic and self-healing infections to disseminated (diffuse CL) or protracted and non-healing (chronic CL) disease (6,7). Both genetic and acquired factors are important in host immunocompetency; malnutrition is an important cause of acquired immunosuppression (8,9). In regards to immunity against leishmaniasis, both protein-calorie deficiency and trace element or vitamin deficiency have been associated in studies with visceral and mucocutaneous leishmaniasis in mice and humans (10–12). To our knowledge, studies regarding the relationship between the clinical courses of CL, in particular, chronic and non-healing cases, and nutritional status are lacking (13). In a previous study, socioeconomic class was shown as a significant indicator in the clinical course of CL. Chronic malnutrition is a consequence of economic and educational deficiency and is a known risk factor for immune deficiency (14).

This study was designed to investigate the relationship between dietary intake of nutrients, including vitamin A, and the clinical course of CL especially in terms of its evolution to chronic forms.

## Materials and Methods

Approval for this study was granted by the Ethics Committee of Mashhad University of Medical Sciences in Imam Reza Hospital, Mashhad, Iran. In this prospective cohort study, 250 cases of leishmaniasis were enrolled from Apr 2011 to Aug 2012. The demographic information of the patients and clinical characteristics of the lesions were recorded. Inclusion criteria were diagnosis of CL, confirmed by Giemsa-stained direct smear, and informed consent. Exclusion criteria were: 1) previously known systemic disease, 2) receiving any kind of systemic immune suppressive therapy or nutritional supplement, 3) being on any type of diet during the previous 6 months,4) unreliability of the responses (including illiteracy and the inability to comprehend the questions). The aims of the study were explained to the participants and they were assured that their information would be kept confidential. Dietary intake was assessed at the beginning of the study. A semi-quantitative 302-item food frequency questionnaire (FFQ) was designed based on two FFQs previously validated in the Iranian population (15,16).This FFQ assessed the subject’s energy, macronutrient, and fiber intake over the previous three days. Pictures of standard portion sizes were used to estimate the usual portions consumed for foods such as rice and pasta. For each food item, the frequency consumed per day was multiplied by the amount consumed, based on portion size, to compute the total amount consumed per day. Iranian food composition tables were used to calculate the daily energy, macronutrient, and fiber intake (17). A trained research assistant explained and administered the FFQ to the patients or their parents. The patients were treated according to the treatment protocols of the leishmaniasis clinic and the clinician's decision; the treatment included systemic and intralesional injection of antimonials and/or cryotherapy. The clinical course of the disease was recorded and the patients received followed-up for at least one year. A control group of 71 individuals including 26 males and 45 females from the family members of the patients, who did not have an active leishmaniasis infection and were clinically healthy, were also enrolled.

To make more meticulous comparisons, patients were divided according to age into two groups: 16 yr and younger and older than 16 yr. The older age group was compared to the control, but the younger group was analyzed in terms of estimated average requirements (EAR).

## Results

From the original 250 patients, only 149 (mean age 21.32±17.62yr) completed this study. The others were lost to follow-up. Sixty-seven (44.9%) were male (mean age 19.35 yr) and 82(55.1%) female (mean age 22.43 yr). The most common location of the leishmaniasis lesion was the face (93), followed by upper arm (26), lower arm (25), and trunk and neck (5). Duration of the leishmaniasis lesions was between 1 to 96 months.

Considering duration of the disease, the patients were divided into two groups: acute (less than a year) and chronic (1 year or longer). Table 1 shows frequency of acute and chronic cases divided by sex.

**Table 1:** Frequency of acute and chronic cases by sex

***Variable***	***Acute***	***Chronic***	***Total***	***Control***
Male(n)	45	22	67	26
%	67.2	32.8	100	
Female(n)	56	26	82	45
%	68.3	31.7	100	
Total(n)	101	48	149	71
%	67.8	32.2	100	

The mean age was 23.76±20.12yr in the acute group and 18.33±15.13yr in the chronic group. Independent samples *t*-test showed no significant relationship between age and course of CL (*P*=0.215).

Of the 73 patients in the older age group, 3 were eliminated because of under-reporting in the food questionnaire (daily energy less than 700 kcal). Among the patients older than 16 yr, the educational level and course of the lesions were negatively associated. In other words, chronic leishmaniasis was more common in patients with lower educational levels (*P*-value of Pearson’s chi-square=0.003).

In the younger age group, 9 (12.2%) were under-weight (less than 5^th^ percentile), 48 (64.9%) were within healthy weight range (5^th^ to 85^th^ percentile),6 (8.1%) were overweight (85^th^ to 95^th^ percentile), and 11 (14.9%) were obese (95^th^ percentile and higher). With the chi-square test, there was no significant relationship between the course of the disease and weight (*P*=0.522). Table 2 shows the comparison of dietary energy and macro and micronutrients between patients older than 16 yr and the control group. Daily in-take of vitamin A was significantly lower among the chronic leishmaniasis group. In addition, we found lower amounts of energy, fiber, and potassium intake in the chronic leishmaniasis group.

Moreover, in comparing the acute and chronic groups with the control group, not only did the chronic leishmaniasis cases have the lowest daily intake of vitamin A, but also other nutrients including energy, fat, protein, fiber, vitamin E, and potassium were significantly lower. When each sex was considered separately, all the differences in the above-mentioned food items persisted in the female groups (acute, chronic and control), but in the male group only daily intake of vitamin A, fat, and fiber and were significantly reduced in the chronic leishmaniasis group. In patients younger than 16 yr only daily intake of vitamin A was significantly different between the acute and chronic cases. Regarding other macro and micronutrients, although daily consumption was lower in the chronic cases, the difference was not statistically significant. Vitamin A consumption was higher than EAR in acute cases but lower than EAR in the chronic case group.

**Table 2:** Comparison of daily intake of macro and micro nutrients in acute and chronic leishmaniasis cases

Variable	Group	Disease course	Mean ± SD	P-value
Energy(Kcal)	Patients	Acute	2176.93±615.231	0.01<
		Chronic	1850.52±353.218	
	Control		2295.30±484.578	
Protein (gm)	Patients	Acute	75.55± 27.496	
		Chronic	67.02± 18.625	0.01
	Control		82.83± 20.475	
Carbohydrate (gm)	Patients	Acute	284.90± 87.160	
		Chronic	246.32± 51.998	0.07
	Control		285.72 ± 70.913	
Fat (gm)	Patients	Acute	80.64 ± 27.245	
		Chronic	65.79± 22.359	>0.001
	Control		91.89± 24.226	
Potassium (mg)	Patients	Acute	2966.60±1054.81	
		Chronic	2402.26±618.26	
	Control		91.89± 24.226	<0.01
Fiber (gm)	Patients	Acute	17.04±7.51	
		Chronic	12.33±4.65	
	Control		20.46±11.31	<0.001
Vitamin A (μg)	Patients	Acute	601.15±300.59	
		Chronic	416.41±180.56	
	Control		855.31±388.46	
Vitamin E (mg)	Patients	Acute	8.56±10.15	<0.001
		Chronic	6.39± 4.41	
	Control		16.96±29.90	
Folate(μg)	Patients	Acute	416.97±353.97	
		Chronic	374.05±321.36	0.001
	Control		386.20±210.09	

## Discussion

Severe malnutrition is probably the most prevalent cause of immunodeficiency worldwide (17). Severe protein-calorie malnutrition influences both humoral and cellular arms of the immune system, modifying and decreasing immune-competent cell proliferation and function, cytokine secretion, and antigen recognition abilities (11,18,19).

Interestingly, leptin, a hormone and cytokine produced mainly by white adipose tissue, is shown to induce protective T helper 1 (Th1) responses. Leptin may have a role in modulation and restoration of effective responses against visceral leishmaniasis (20). A handful of animal studies on mice demonstrated that in malnutrition, in parallel to the dietary reduction in protein, zinc, and iron, an increase in splenic *Leishmania* parasite load, reduced production of INF γ by spleen cells, and depression of immune responses occurs (12, 21).

Moreover, higher arginase expression of monocytes and macrophages of malnourished mice has been linked to a more permissive environment for *Leishmania* growth (22), likewise, serum levels of vitamin A and other nutritional indices were lower in the children with visceral leishmaniasis than their non-infected relatives in a study (23). Regarding CL, in Iran, the Zn/Cu ratio and serum levels of zinc and iron were significantly lower in patients as compared to the controls (24). Contrastingly, another study from Bolivia compared blood levels of calcium, magnesium, sodium, potassium, phosphate, lipids, vitamin B12, folate, iron, ferritin, TIBC, and albumin in CL patients with control group and concluded that nutritional status of patients was essentially normal (25). Moreover, serum albumin depletion has been linked to delay healing of the CL lesions (26).

Although Vitamin A deficiency may impair both the Th1and type 2 cells (Th2) immune responses, Th1 responses are principally affected. Vitamin A deficiency at the time of exposure to antigen may increase the development of interleukin 10 (IL-10) producing Th2 and regulatory cells, and decrease the development of Th1 memory cells (27). These immunologic pathways are critical to both effectively control leishmaniasis and prevent chronicity. Low levels of Vitamin A in the chronic cases in this study were remarkable; this can be explained on the basis of polarization of immune responses toward Th2 in a deficiency state that leads to a non-healing clinical course.

In our study, the lower intake of protein and energy in chronic leishmaniasis cases is also consistent with a recent murine study. That study showed that in malnourished mice high arginase activity and normal nitric oxide production capacity of monocytes and macrophages provide a more permissive environment for *Leishmania* parasite growth (28). In other studies, malnutrition has been associated with a relative increase of anti-inflammatory prostaglandin over pro-inflammatory leukotriene production by tissue macrophages (29).

Folate plays an essential role in nucleic acid and protein synthesis. In the deficiency states, proliferation of T lymphocytes to mitogen activation and proportion of circulating T lymphocytes were diminished (30).

Since free radicals are immunosuppressive, vitamin E with its strong anti-oxidant activity is considered an immune response enhancer. Vitamin E supplementation in healthy adults significantly increased T cell population and improved CD4/CD8 (29). Regarding carbohydrate, fat, fiber, and potassium deficiency, they are only coincident to a general malnutrition state.

## Conclusion

Dietary supplementation may improve host response to the *Leishmania* parasite and reduce the probability of chronicity in the course of CL.

## Ethical considerations

Ethical issues (Including plagiarism, informed consent, misconduct, data fabrication and/or falsification, double publication and/or submission, redundancy, etc.) have been completely observed by the authors.
